# Design of a Sub-Picosecond Jitter with Adjustable-Range CMOS Delay-Locked Loop for High-Speed and Low-Power Applications

**DOI:** 10.3390/s16101593

**Published:** 2016-09-28

**Authors:** Bilal I. Abdulrazzaq, Omar J. Ibrahim, Shoji Kawahito, Roslina M. Sidek, Suhaidi Shafie, Nurul Amziah Md. Yunus, Lini Lee, Izhal Abdul Halin

**Affiliations:** 1 Department of Electrical and Electronic Engineering, Faculty of Engineering, Universiti Putra Malaysia, Serdang 43400, Selangor, Malaysia; omar.j.ibrahim@gmail.com (O.J.I.); roslinams@upm.edu.my (R.M.S.); suhaidi@upm.edu.my (S.S.); amziah@upm.edu.my (N.A.M.Y.); izhal@upm.edu.my (I.A.H.); 2 Department of Electronic and Communications Engineering, Al-Nahrain University, Al-Jadriya Complex, Baghdad 10070, Iraq; 3 Imaging Devices Laboratory, Research Institute of Electronics, Shizuoka University, 3-5-1 Johoku, Nakaku, Hamamatsu, Shizuoka 432-8011, Japan; kawahito@idl.rie.shizuoka.ac.jp; 4 Faculty of Engineering, Multimedia University, Persiaran Multimedia, Cyberjaya 63100, Malaysia; linilee@mmu.edu.my

**Keywords:** delay step, delay range, time jitter, Delay-Locked Loop (DLL), charge pump, Capacitor-Reset Circuit (CRC)

## Abstract

A Delay-Locked Loop (DLL) with a modified charge pump circuit is proposed for generating high-resolution linear delay steps with sub-picosecond jitter performance and adjustable delay range. The small-signal model of the modified charge pump circuit is analyzed to bring forth the relationship between the DLL’s internal control voltage and output time delay. Circuit post-layout simulation shows that a 0.97 ps delay step within a 69 ps delay range with 0.26 ps Root-Mean Square (RMS) jitter performance is achievable using a standard 0.13 µm Complementary Metal-Oxide Semiconductor (CMOS) process. The post-layout simulation results show that the power consumption of the proposed DLL architecture’s circuit is 0.1 mW when the DLL is operated at 2 GHz.

## 1. Introduction

Delay-Locked Loops (DLLs) with high-resolution delay steps are extensively used for time management of large systems [1]. For example, they are used in Fluorescence Lifetime Imaging Microscopy (FLIM) sensors where a light pulse is modulated with the capture window that is shifted in picosecond-order delay steps for a total range of tens of picoseconds [2]. Furthermore, high-resolution DLLs are used in the compensation for PVT variations and any delay mismatch that may be caused to signals during the operation of many high-frequency VLSI circuits [3]. For all of these applications, DLLs should generate an adequate amount of lock/delay range while maintaining the output jitter as low as possible. This is because there is a trade-off relation between delay range and the jitter performance [4]. In addition, the total delay fluctuations including jitter should be less than the delay resolution for optimum operation [5].

Since DLLs only adjust the phase (delay) of an input signal and not its frequency, DLLs suffer from limited delay range. Therefore, a considerable amount of new techniques has been developed to address this issue. For example, a technique employing a Digital-to-Analog Converter (DAC) with Parallel Variable Resistor (PVR) is used to realize high-resolution delay steps with a wide delay range by accurately controlling the Current-Controlled Delay Element (CCDE) of the DLL [1]. Another technique developed is the use of a dual-loop architecture which utilizes multiple delay lines [6]. The first “reference” loop generates a clock with quadrature phases. In the second “main” loop, these phases are delayed by four Voltage-Controlled Delay Lines (VCDLs) and then multiplexed to generate the output clock. A new technique based on cycle-controlled delay unit was proposed by [7] to enlarge the delay range by reusing the delay units in a cycle-like process without the need for cascading a large number of delay units. A DLL with a new voltage-controlled delay element based on body-controlled current source and body-feed technique was also developed to widen the delay range [8]. In this method, the Phase Detector (PD) is replaced by a Phase/Frequency Detector (PFD) with a start controller to achieve a sufficient locking range. Another new architecture was proposed by [9] which employs a mixed-mode Time-to-Digital Converter (TDC) for enabling a frequency-range selector. The frequency-range selector can generate digital control signals to switch the delay range of the multi-controlled delay cell in the VCDL and the current of the digitally-controlled charge pump.

However, the majority of the techniques mentioned above result in complex circuit architectures that lead to degraded jitter performance as well as increased area overhead, cost, and power consumption. Motivated by this research gap, this work proposes a new and simple technique using a Capacitor-Reset Circuit (CRC) to reset the loop filter capacitor for delay range extension and at the same time reducing the jitter performance into the sub-picosecond range. The capacitor-reset technique is widely used to reinitialize a control voltage to a fixed initial value and has been applied in many circuits such as pixels of image sensors and PLL circuits [10,11]. At this point, mathematical analysis confirmed by circuit simulation, our proposed technique is capable of generating a comparably wide delay range and picosecond-resolution delay steps with a sub-picosecond jitter performance. In addition, this architecture consumes a relatively small area and power compared with the available techniques reported in literature.

Table 1 below shows performance specifications of the most recent and relevant high-resolution DLL designs reported in the literature.

Table 1 summarizes the DLL’s parameters that have a direct impact on the performance in terms of speed and power consumption. For example, information about achievable delay range, delay resolution, number of delay steps, operating frequency range, RMS jitter, and power consumption is provided in Table 1. It is worth noting that the finest delay step is 4 ps achieved by [12]. It also generates a comparably long delay range of approximately 345 ps.

The proposed design is explained in the subsequent section. The results and discussion are presented in Section 3. Finally, Section 4 summarizes and concludes this paper.

## 2. Materials and Methods

**Figure 1:**
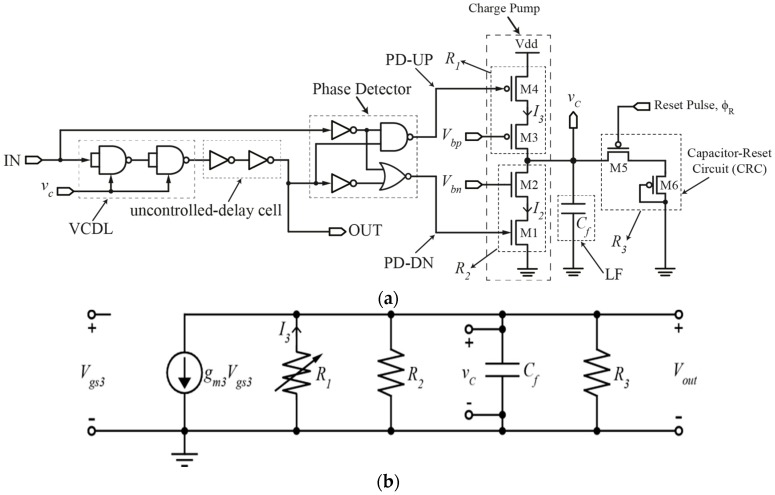
(**a**) Schematic of DLL with the CRC; and (**b**) small-signal model of DLL’s charge pump with CRC.

Our proposed circuit is shown in Figure 1a. It consists of a conventional VCDL, an Exclusive-OR (XOR) gate-based Phase Detector (PD), a Charge Pump (CP), and a modified Loop Filter (LF) with the addition of the CRC. It works by resetting the loop filter’s capacitor by a pre-determined time constant before lock is achieved. The reset operation is performed by a reset signal, ϕ_R_. By varying the pulse width of ϕ_R_ using a simple Pulse-Width Generator (PWG) circuit that will be illustrated at the end of this section, a change in the time constant *τ_R_* of the modified loop filter is achieved. This results in a change in delay range of the DLL.

Figure 1a shows the modification made to the loop filter of a DLL where M5 and M6 are used to create the CRC. M5 acts as a switch that resets the loop filter’s capacitor, *C_f_*. On the other hand, M6 is connected as a diode whose resistance together with the capacitance *C_f_* of the loop filter’s capacitor creates a time constant *τ_R_* that controls the magnitude of *v_c_* which is fed to the VCDL current to control bias current and propagation delay. This ultimately controls the delay step and lock/delay range. The aspect ratio of both pMOS transistors, M5 and M6, is 0.35 μm/0.13 μm.

The capacitor-reset operation at a pre-determined reset signal duration results in a varying charge/discharge rate of *C_f_* when *V_bp_* is changed as opposed to a DLL without the CRC. This results in changes in *v_c_* settling time that controls the delay of the VCDL. To illustrate this operation, the charge pump’s small-signal model shown in Figure 1b is used. *v_c_* is expressed in Equations (1) and (2) during charging and discharging operations, respectively.

(1) $$v_{c}(t)=v_{ch0}\left(1-e^{-\frac{t}{\tau_{R}}}\right),$$

(2) $$v_{c}(t)=v_{dis0}\left(e^{-\frac{t}{\tau_{R}}}\right).$$

where *v_ch0_*, *v_dis0_*, and *τ_R_* are the initial voltage across *C_f_* during charging (which is equal to −*V_Tp_*), initial voltage across *C_f_* during discharging (which is equal to maximum *v_c_*), and the time constant of the loop filter, respectively. This time constant, *τ_R_*, is written as:

(3) $$\tau_{R}=R_{3}C_{f}.$$

where *R*_3_ is the equivalent resistance of the diode-connected transistor’s (M6) output resistance in series with transistor M5’s output resistance.

From Equations (1) and (2), it is obvious that the capacitor’s voltage *v_c_* is directly dependent on charging/discharging time, *t*. Equations (1) and (2) also implies that the charging/discharging time of *C_f_* can be changed by changing the time constant *τ_R_* of the CRC, which will in turn change *v_c_*. This is achieved through changing the reset duration of the reset signal ϕ_R_ that is applied to the gate of M5 (see Figure 1a).

The small-signal model of the charge pump connected to the CRC shown in Figure 1b is also used to analyze how the DLL generates fine-linear delay steps within a selectable delay range. *V_bp_* is varied in order to vary the delay steps. The series output resistance of transistors M4 and M3 is modeled as *R*_1_ in Figure 1b. Likewise, *R*_2_ in Figure 1b models the series output resistance of transistors M2 and M1 and *R*_3_ models the output resistance of the diode-connected transistor M6 in series with transistor M5’s output resistance when M5 is turned on. It should be mentioned that the aspect ratio of the nMOS transistors M1 and M2 is 0.6 μm/0.13 μm, and that of the pMOS transistors M3 and M4 is 1.2 μm/0.13 μm. The value of the capacitance *C_f_* in Figure 1a,b is 0.63 fF.

*R*_1_, *R*_2_ and *R*_3_ are given by Equations (4)–(6), respectively [16]:

(4) $$R_{1}\approx \left(g_{m3}r_{ds3}\right)r_{ds4}.$$

(5) $$R_{2}\approx \left(g_{m2}r_{ds2}\right)r_{ds1}.$$

(6) $$R_{3}\approx \left(g_{m5}r_{ds5}\right)r_{ds6}.$$

**Figure 2:**
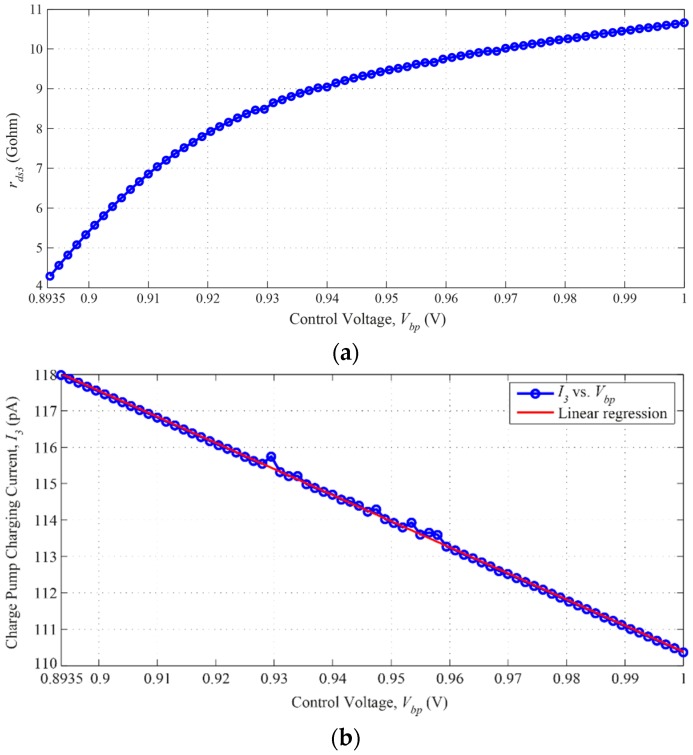
(**a**) *r_ds3_* versus control voltage, *V_bp_*; and (**b**) *I*_3_ versus control voltage, *V_bp_*.

When *V_bp_* is varied, it is obvious that *r_ds3_* and *g_m3_* change accordingly, resulting in a change in *R*_1_. Due to this, *R*_1_ is written as:

(7) $$R_{1}\approx \left(g_{m3}+\Delta g_{m3}\right)\left(r_{ds3}+\Delta r_{ds3}\right)r_{ds4},\;R_{1}\approx g_{m3}r_{ds3}r_{ds4}+r_{ds4}\left(g_{m3}\Delta r_{ds3}+r_{ds3}\Delta g_{m3}+\Delta r_{ds3}\Delta g_{m3}\right).$$

where *∆r_ds3_* and *∆g_m3_* are the changes in *r_ds3_* and *g_m3_*, respectively. Equation (7) represents the change in *R*_1_ when *V_bp_* ≠ 0 and can also be written in the following form:

(8) $$R_{1}\approx R_{C_{0}}+R_{P}.$$

where *R_C0_* is a constant corresponding to the term (*g_m3_r_ds3_r_ds4_*) and *R_P_* is a variable corresponding to the term (*r_ds4_(g_m3_∆r_ds3_ + r_ds3_∆g_m3_ + ∆r_ds3_ ∆g_m3_*)) in Equation (7). According to simulation results shown in Figure 2, when *V_bp_* is varied from 1 V to 0.8935 V, *Δr_ds3_* changes from 10.66 GΩ to 4.29 GΩ. Likewise, for the same *V_bp_* range, the charge pump’s charging current *I_3_* changes from 110.37 pA to 117.99 pA. However, when *V_bp_* = 0, *r_ds3_* and *g_m3_* are at their minimum values. This implies that *∆r_ds3_* and *∆g_m3_* will have very small values which can be neglected compared with other *R*_1_ cases in which *V_bp_* ≠ 0. Hence, Equation (8) can be rewritten as:

(9) $$R_{1,0}\approx R_{C_{0}}.$$

where *R*_1,0_ represents the case when *V_bp_* = 0. Thus, Equation (8) can be rewritten as follows:

(10) $$R_{1}\approx R_{1,0}+R_{P}.$$

It should be mentioned that the non-monotonicity points in Figure 2b can be a consequence of the non-convergence problems. These problems can be caused during the simulation if the resistance of the transistor is very high or very low. This can be solved by adjusting either the simulator options or the transistor model parameters (Ron and/or transconductance *g_m_*) [17]. However, no significant impact can be observed in the overall behavior and performance of the DLL circuit, as will be demonstrated in the delay steps linearity results explained in the next section. In addition, a linear regression has been employed and superimposed on the plot in Figure 2b regarding the charge pump charging current *I*_3_ versus the control voltage *V_bp_*. It can be seen from Figure 2b that the Root-Mean Square Error (RMSE) of the linear regression plot is only 0.06, which indicates that the original plot of *I*_3_ versus *V_bp_* is almost linear.

According to [1], the relationship between the change in resistance and the change in current is expressed as:

(11) $$\frac{R_{1}\left(t-1\right)}{R_{1}\left(t-1\right)+\Delta R_{1}\left(t\right)}\approx \frac{I_{3}\left(t-1\right)-\Delta I_{3}\left(t\right)}{I_{3}\left(t-1\right)}.$$

The time delay, *t_d_*, of the VCDL is given as [18]:

(12) $$t_{d}=T_{ref}-\left(K_{VCDL}v_{c}\right)+\Delta d.$$

where *T_ref_*, *K_VCDL_*, and *∆d* are the period time of the input clock signal, the gain of the VCDL, and the jitter caused by the VCDL, respectively. Equation (12) indicates that the voltage, *v_c_*, across the capacitor determines the time delay, *t_d_*. The voltage *v_c_* can be written as [18]:

(13) $$v_{c}\left(t\right)=\frac{1}{C_{f}}\int_{0}^{\Delta t}I_{3}\left(t\right)\times dt+v_{c}\left(0\right),\;v_{c}\left(t\right)=\frac{1}{C_{f}}\int_{0}^{\Delta t}I_{3}\left(t\right)\times dt+\left(-V_{Tp}\right).$$

Substituting Equation (13) into Equation (12), *t_d_* is written as:

(14) $$t_{d}=T_{ref}-\left(K_{VCDL}\times \left(\frac{1}{C_{f}}\int_{0}^{\Delta t}I_{3}\left(t\right)\times dt+\left(-V_{Tp}\right)\right)\right)+\Delta d.$$

Substituting Equation (11) for *I*_3_ into Equation (14) yields *t_d_* in terms of the change in *R*_1_ and *I*_3_ and is written as:

(15) $$t_{d}=T_{ref}-\left(K_{VCDL}\times \left(\frac{1}{C_{f}}\int_{0}^{\Delta t}\frac{\left(R_{1}\left(t-1\right)\right)\times \left(I_{3}\left(t-1\right)\right)}{R_{1}\left(t-1\right)+\Delta R_{1}\left(t\right)}\times dt+\left(-V_{Tp}\right)\right)\right)+\Delta d.$$

*t_d_* in Equation (15) represents the delay step. On the other hand, the delay range, *t_dr_*, is defined as the difference between maximum and minimum delays and can be written as:

(16) $$t_{dr}=t_{d(max)}-t_{d(min)}.$$

where *t_d(max)_* and *t_d(min)_* are the maximum and the minimum delays. On the other hand, the maximum and minimum delays are expressed by Equations (17) and (18), respectively:

(17) $$t_{d(max)}=T_{ref}-\left(K_{VCDL}\times \left(\frac{1}{C_{f}}\int_{0}^{\Delta t\;max}\frac{\left(R_{1}\left(t-1\right)\right)\times \left(I_{3}\left(t-1\right)\right)}{R_{1}\left(t-1\right)+\Delta R_{1(max)}\left(t\right)}\times dt+\left(-V_{Tp}\right)\right)\right)+\Delta d_{max},$$

(18) $$t_{d(min)}=T_{ref}-\left(K_{VCDL}\times \left(\frac{1}{C_{f}}\int_{0}^{\Delta t\;min}\frac{\left(R_{1}\left(t-1\right)\right)\times \left(I_{3}\left(t-1\right)\right)}{R_{1}\left(t-1\right)}\times dt+\left(-V_{Tp}\right)\right)\right)+\Delta d_{min}.$$

**Figure 3:**
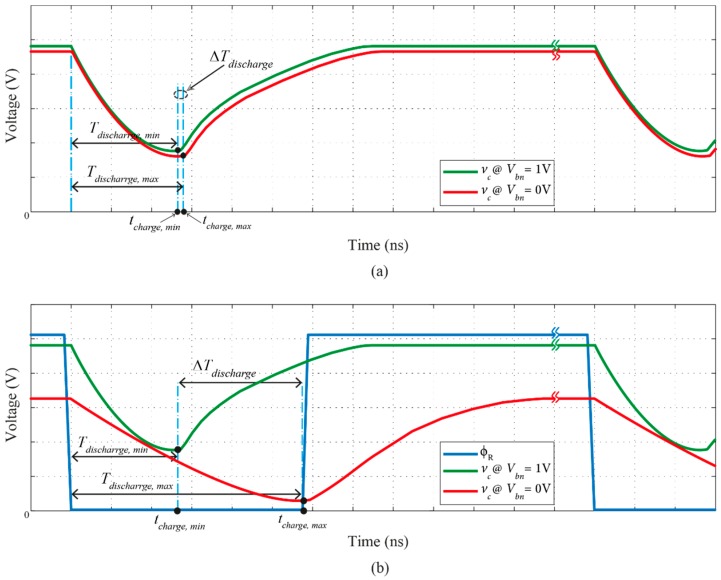
Difference in discharge rates for two cases: (**a**) without CRC; and (**b**) with CRC.

In order to demonstrate the operation of the charge pump circuit without and with the proposed CRC technique, Figure 3 is considered. This figure is an illustration figure that illustrates the differences in the discharge rates for two extreme values of *V_bn_* (1 V and 0 V). Figure 3a highlights the discharge rates for a charge pump without the proposed CRC and Figure 3b with the CRC. It is obvious from Figure 3b that the difference in discharge rates is significantly higher than that of the case in Figure 3a. To clarify this, according to the simulations, the discharge rates’ difference for the case with the CRC technique is 2.49 mV/ps, while that for the case without CRC is only 0.2 mV/ps. The higher is the difference in the discharge rate, the bigger is the difference in *v_c_* settling values according to Equations (1), (2), (15), (17) and (18). In relation to the simulation results, the discharge rate of *v_c_* is faster when *V_bn_* value is 1 V, causing the capacitance *C_f_* to fully discharge faster and the discharging time to have a lower value compared to the case when *V_bn_* is 0 V. The discharge rate is directly proportional to the discharge current *I_2_*. Since the discharge rate is different, the settling voltage for *v_c_* is also different, causing a change in the control voltage of the VCDL and resulting in a change in time delay of the DLL.

**Figure 4:**
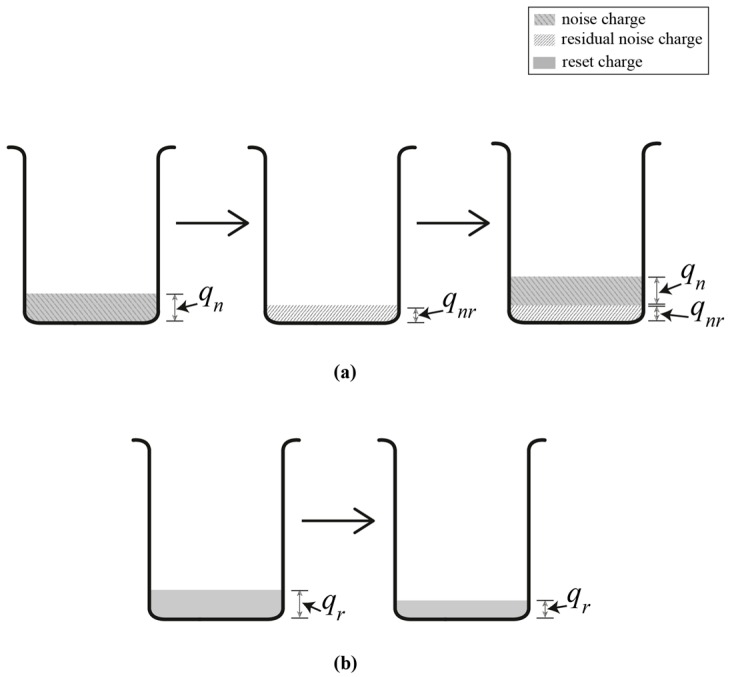
Error charge accumulation in loop filter’s capacitor: (**a**) without CRC; and (**b**) with CRC.

The charge pump circuit of a DLL suffers from amplifier noise charge injected from its amplifier into the loop filter capacitor, thus reducing this noise will result in better time jitter performance [19]. Figure 4 is used to illustrate error charge accumulation in charge pump’s loop filter capacitor. Figure 4a shows a conventional charge pump where initially amplifier noise charge *q_n_* is injected into *C_f_* from the amplifier at the ON phase of the input signal. When the input signal goes low, *C_f_* discharges but a small amount of residual noise charge *q_nr_* is left in C_f_. The next ON phase of the input signal injects new amplifier noise charge and it is added with the residual noise charge left from the previous discharge cycle. Therefore, for simplicity, the output voltage *v_c_* of the charge pump, is given by:

(19) $$v_{c}=\frac{q_{n}+q_{nr}}{C_{f}}.$$

The numerator of Equation (19) gives the total noise charge of a conventional charge pump. On the other hand, Figure 4b shows that noise charge is also transferred into the loop filter's capacitor. However, when the signal goes high, the CRC is activated and causes *C_f_* to fully discharge. Only a small amount of reset charge *q_r_* is injected into *C_f_* from transistors M5 and M6 that make up the CRC (see Figure 1a). Thus, the output voltage *v_c_* of the charge pump is given by:

(20) $$v_{c}=\frac{q_{r}}{C_{f}}.$$

We can also view *q_r_* as noise charge since it is random in nature. However, *q_r_* is much less than *q_n_* + *q_nr_*, thus the jitter of *v_c_* is significantly reduced for a charge pump with the CRC. Moreover, this technique also produces a wider delay range since a larger and accurate level of *v_c_* can be achieved when the ON phase period of the reset signal ϕ_R_ is made longer through the PWG circuit.

**Figure 5:**
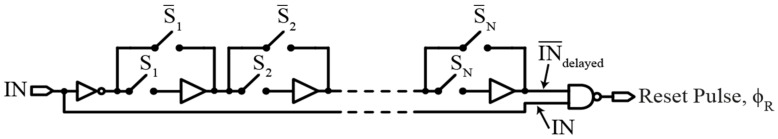
Simple Pulse-Width Generator (PWG) circuit.

The PWG circuit, used to control the pulse width of the reset signal ϕ_R_, is shown in Figure 5. This circuit is used to set the time constant of the loop filter in order to set the desired discharge rate of *C_f_* (see Equations (1) and (2)). Once a desired delay range is acquired, the charge pump’s charging current *I*_3_ can be varied through the charge pump amplifier’s bias voltage, *V_bp_* (see Figure 1a), to allow small-linear changes in the DLL’s output signal time delay.

**Figure 6:**
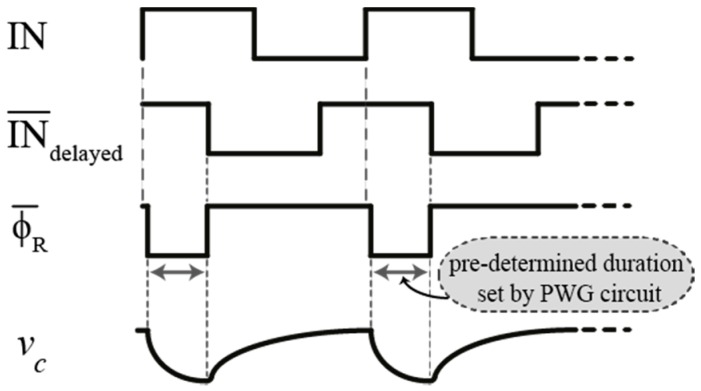
Timing diagram showing how the reset signal activation is synchronized with the input reference pulse.

It is also noted that the input of the PWG circuit shown in Figure 5 is fed from the input signal of the DLL itself in order to synchronize the discharge time of node *v_c_* with the input pulse, as illustrated in the timing diagram shown in Figure 6.

## 3. Results and Discussion

The proposed DLL is simulated using a 0.13 µm CMOS process. The power supply voltage is 1.2 V. From post-layout simulations, the delay is controlled from zero to 69 ps by varying *V_bp_* from 0.8935 V to 1 V in steps of 1.5 mV. In this simulation, parametric analysis was used to change *V_bp_*; however, the value of *V_bp_* can be controlled by a 10-bit Digital-to-Analog Converter (DAC). *V_bn_* is fixed at 0.2 V.

**Figure 7:**
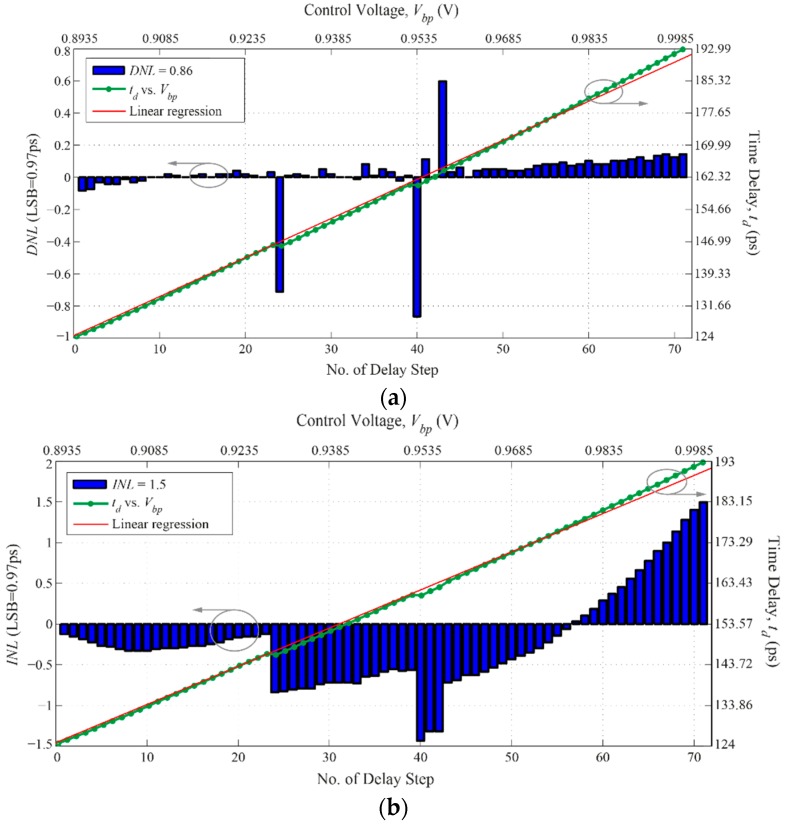
DLL’s total time delay and control voltage ranges with respect to the delay steps linearity: (**a**) *DNL*; and (**b**) *INL*.

Figure 7 shows the generated output time delay *t_d_* as a function of the control voltage *V_bp_* with respect to the delay steps linearity. It is clear from Figure 7 that the time delay increases linearly with the increase in *V_bp_*. The sensitivity of the linear regression plot is approximately 644 (ps/V) with Root-Mean Square Error (RMSE) equals to 0.64. For an LSB of 0.97 ps, it can be seen in Figure 7a that the delay steps’ *Differential Non-Linearity* *(DNL)* does not exceed 0.86. Moreover, the *DNL* values of the delay steps located between the 41st and 70th delay steps are all concentrated in the positive region. This has mainly caused the slight deviation observed between the linear regression and the simulated output delay steps shown in Figure 7a,b, and it has also resulted in the maximum 1.5 *Integral Non-Linearity* (*INL*) value at the end of the *INL* plot in Figure 7b. On the other hand, the *INL* values across the generated delay steps in Figure 7b are somewhat concentrated in the negative region. This indicates that the resolution of most of the delay steps is very close to one LSB of the output delay.

**Figure 8:**
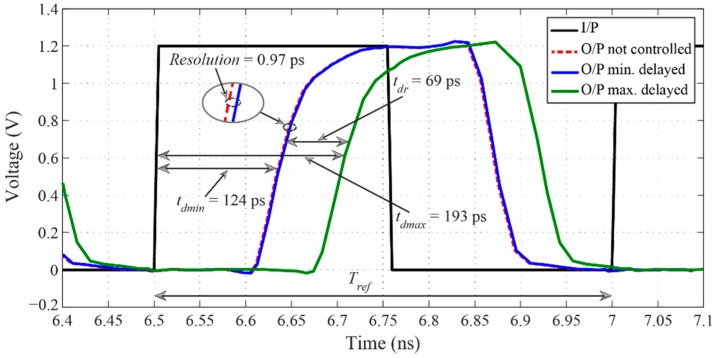
DLL’s input and output signals when operated at 2 GHz of the input reference signal, where *t_dmin_* and *t_dmax_* correspond to the minimum and maximum times of the output time delay *t_d_* and their values are 124 ps and 193 ps, respectively, the loop filter’s time constant *τ_R_* values are 2.32 µs at *t_dmin_* and 14.22 µs at *t_dmax_*, and the reference signal’s period *T_ref_* is 0.5 ns.

Figure 8 shows the simulated DLL’s output signal, which is delayed by 0.97 ps as the minimum delay step and total of 69 ps as the maximum delay range, when operated at 2 GHz of the input reference signal. The lock-in time of the DLL is only 14 cycles.

**Figure 9:**
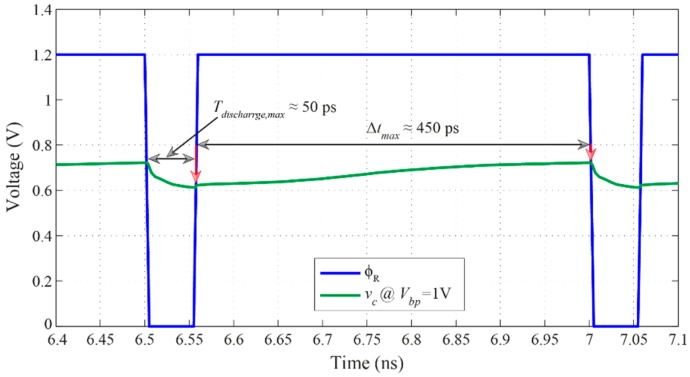
Voltage across the loop filter’s capacitor *v_c_* and reset signal ϕ_R_ at locked state.

Figure 9 shows the simulated voltage *v_c_* across the loop filter’s capacitor with the reset signal ϕ_R_.

**Figure 10:**
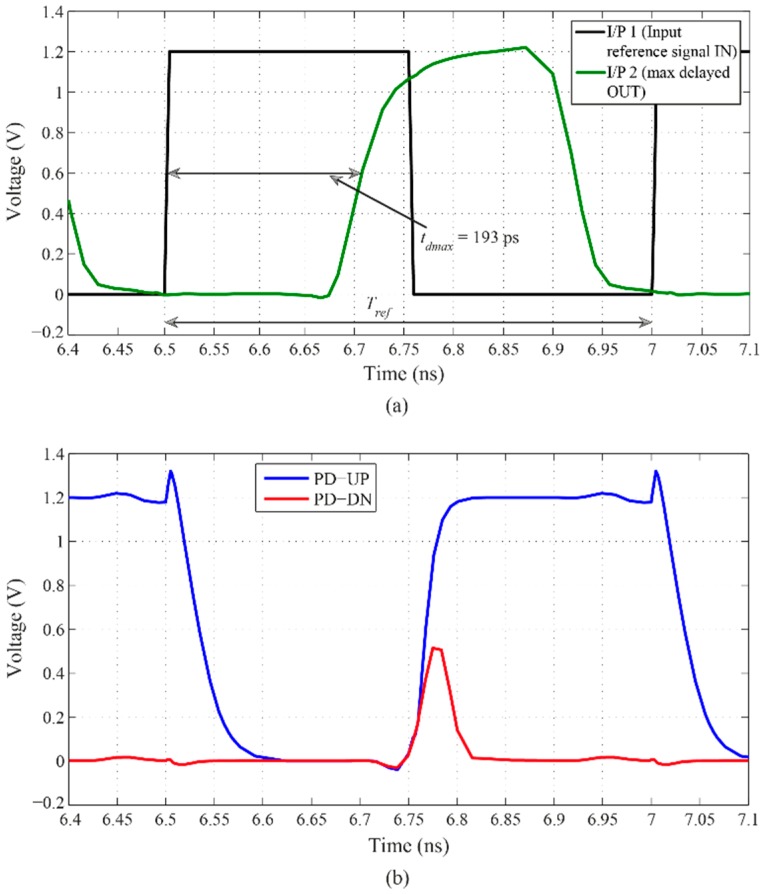
Input and output signals of the phase detector at locked state: (**a**) Inputs; and (**b**) Outputs.

It can be seen in Figure 9 that the duration of the reset signal ϕ_R_ is almost identical to the maximum discharge time, *T_discharge,max_*, obtained when *V_bp_* equals to 1 V. The waveforms plotted in Figure 9 have been obtained after the locked state has been achieved, i.e., after 14 cycles. Likewise, at locked state and when *V_bp_* equals to 1 V, the input and output signals of the phase detector are presented in Figure 10. Figure 10a shows the input reference and output delayed signals which are fed to the two inputs of the phase detector. According to the phase difference between these two inputs, the phase detector generates phase difference information, signal “PD-UP” and signal “PD-DN” in Figure 10b, which is fed to the charge pump to keep the operation of the DLL in the locked state.

**Figure 11:**
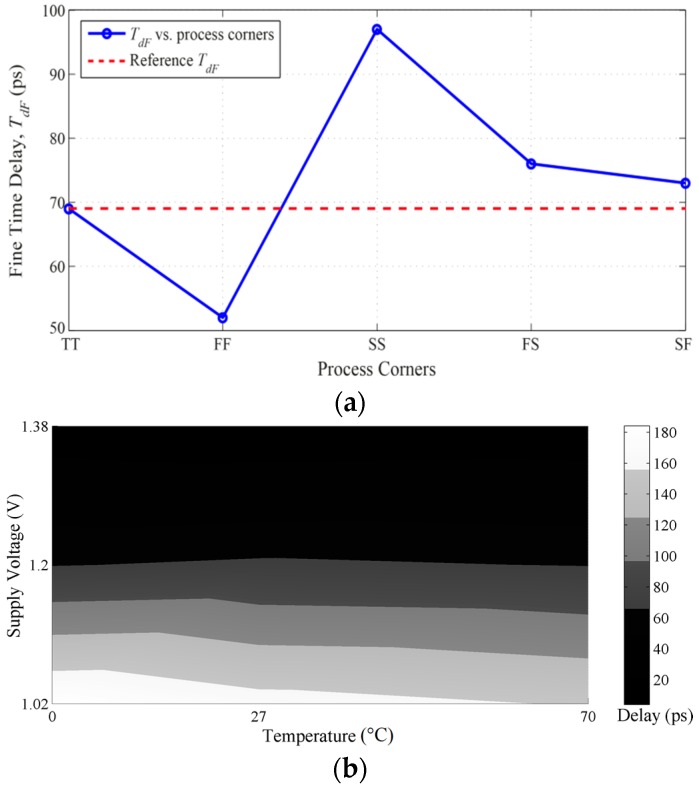
Maximum DLL’s delay range versus: (**a**) process corners; and (**b**) temperature and supply voltage variations.

For completeness, the PVT variations effects on the DLL’s delay range have been simulated and analyzed, as shown in Figure 11. Since the maximum achievable delay range is 69 ps, it can be noted in Figure 11a that the process corner FF can degrade the delay range and the corner SS can mostly degrade the jitter through the extremely extended range. Nonetheless, extending or narrowing the pulse width of the reset pulse, ϕ_R_, can solve these shortcomings. In Figure 11b, three temperature and voltage variations all located at 1.38 V for 0 °C, 27 °C, and 70 °C, which are all dark black-colored, can degrade the delay range by 12 ps, 15 ps, and 18 ps, respectively. Similarly, the small violations in the delay range with the other PVT variations can be compensated by extending the pulse width of ϕ_R_ without significantly degrading the total output jitter or the delay steps linearity.

**Figure 12:**
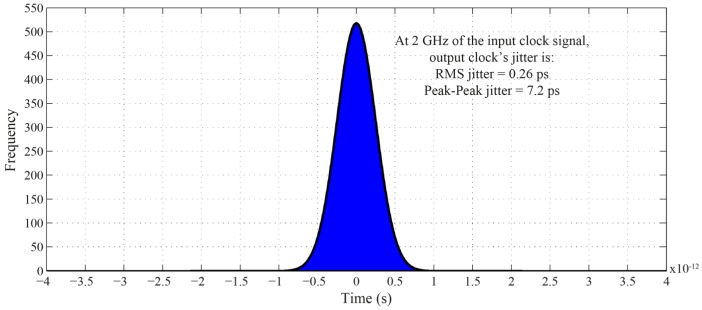
DLL output jitter histogram.

Simulation results on jitter show that the output jitter of the DLL is remarkably low. Figure 12 shows the simulated jitter when the DLL is operated at 2 GHz of the input reference signal. The peak-to-peak and RMS values are 7.2 ps and 0.26 ps, respectively. As mentioned, this is attributed to the cycle-to-cycle reset operation of the charge pump’s capacitor, which significantly minimizes the accumulated noise originated from the charge pump’s amplifier. It is also worth mentioning that the low jitter is attributed to the use of only one NAND gate-based buffer in the VCDL circuit.

**Figure 13:**
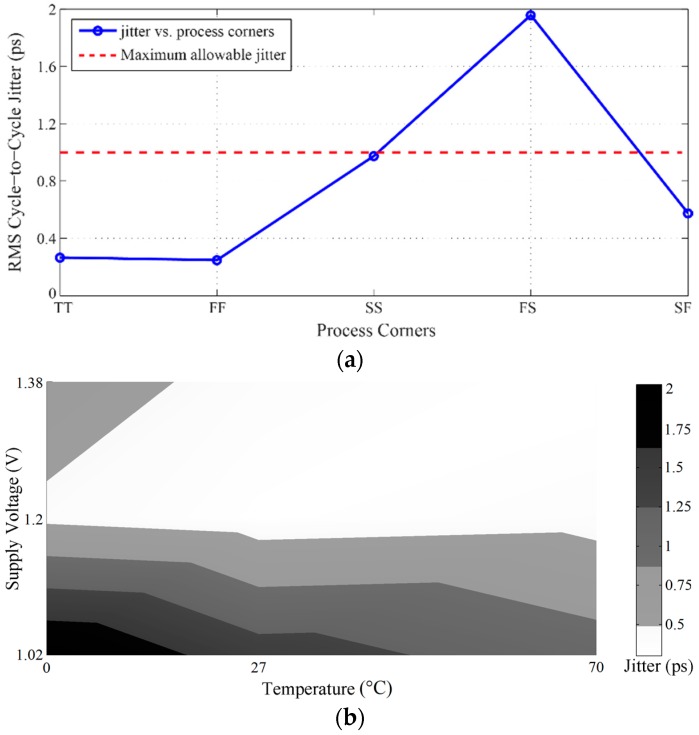
RMS cycle-to-cycle jitter versus: (**a**) process corners; and (**b**) temperature and supply voltage variations.

In addition, the effects of the PVT variations on the jitter performance have also been simulated and analyzed, as shown in Figure 13.

Since the desired value of the output jitter is in the sub-picosecond range, it can be noted in Figure 13a that only the process corner FS degrades the jitter. However, this shortcoming can be mitigated by optimizing the pulse width of the reset pulse, ϕ_R_. In Figure 13b, only two temperature and supply voltage variations located at 1.02 V for 0 °C and 27 °C, which are dark black-colored and dark grey-colored, can degrade the output jitter to over 1.75 ps RMS and 1.3 ps RMS, respectively.

A summary of the performance specifications and results of the proposed work is presented in Table 2. The proposed CRC technique successfully achieves sub-picosecond-resolution delay step, a high number of delay steps within a specific range, sub-picosecond jitter performance, a wide operating frequency range, sub-milliwatt power consumption, and a small occupied active area for layout. The layout area is significantly minimized because the VCDL, which is followed by an uncontrolled inverter-based buffer as shown in Figure 1a, only uses a single NAND-based buffer. It is worth mentioning that the achieved delay range for the case without the proposed CRC technique is only 2 ps using the same transistor sizes and operating conditions as in the case with the CRC whose achieved delay range is 69 ps.

**Figure 14:**
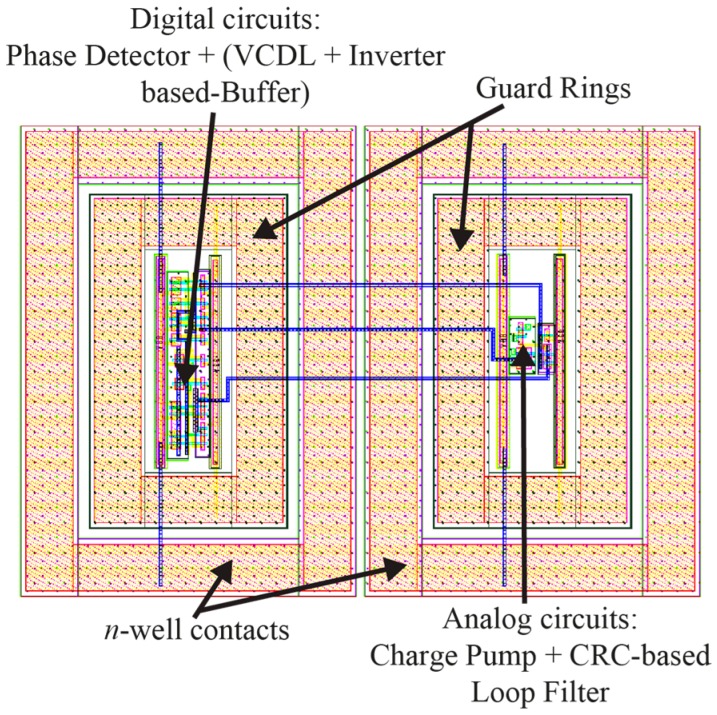
Layout of the proposed DLL.

The layout of the proposed DLL circuit architecture is shown in Figure 14.

It can be seen in Figure 14 that guard rings and *n*-well contacts have been used for the proposed DLL’s layout in order to reduce the effects of the substrate and power noise. In addition, separation of the digital circuits from the analog circuits as well as utilizing separate VDD and GND lines for each of these circuits have been employed to further reduce the substrate noise effects.

In order to compare the performance of this work with other reported high-resolution DLL circuits, Table 3 is presented. In this table, the proposed work has been compared with the work reported by [12], which has been presented earlier in Table 1 and has shown to have almost the best performance compared with the other works in Table 1.

According to Table 3, the proposed lock-range extension technique in this work achieves higher-resolution delay step, higher number of delay steps within a specific range, better jitter performance, lower power consumption, and smaller occupied active area.

## 4. Conclusions

The proposed DLL architecture uses a CRC at the output of the DLL’s charge pump in order to change the delay range and generate small steps with sub-picosecond jitter performance. Through simulation, the DLL maximum delay is 69 ps with 0.97 ps delay steps, while maintaining the total jitter at the output in the sub-picosecond range. In terms of circuit complexity, our proposed technique is much simpler when compared to others as only a reset circuit is added to the charge pump. This not only allows a smaller layout area, but also enhances the DLL’s jitter performance and output range.

## Figures and Tables

**Table 1:** Performance specifications of previously reported high-resolution DLLs.

Variable	[12]	[13]	[14]	[15]
CMOS technology	130 nm	55 nm	350 nm	65 nm
Supply voltage	1.5 V	1 V	3.5 V	1 V
Delay range	345 ps	128 ps	375 ps	161 ps
Delay resolution	4 ps	8.5 ps	7.5 ps	5.21 ps
No. of steps	63	15	7	31
Operating frequency range	1.5–2.5 GHz	200–850 MHz	N/A	3 MHz–1.8 GHz
RMS jitter	N/A	0.04 ps @ 850 MHz	7.5 ps @ 400 MHz	0.85 ps @ 1.8 GHz
Power consumption	30 mW @ 2.5 GHz	1.02 mW @ 850 MHz	N/A	9.5 mW @ 1.8 GHz
Active area	0.03 mm^2^	0.007 mm^2^	N/A	0.0153 mm^2^

**Table 2:** Summary of performance specifications and results achieved by the proposed DLL design.

Variable	Value
CMOS technology	130 nm
Supply voltage	1.2 V
Delay range	69 ps
Delay resolution	0.97 ps
No. of steps	71
Operating frequency range	50 MHz–2 GHz
RMS jitter	0.26 ps @ 2 GHz
Power consumption	0.1 mW @ 2 GHz
Active area	0.001 mm^2^

**Table 3:** Performance comparison of this work with a reported high-resolution DLL.

Variable	[12]	This Work
CMOS technology	130 nm	130 nm
Supply voltage	1.5 V	1.2 V
Delay range	345 ps	69 ps
Delay resolution	4 ps	0.97 ps
No. of steps	63	71
Operating frequency range	1.5–2.5 GHz	50 MHz–2 GHz
RMS jitter	N/A	0.26 ps @ 2 GHz
Power consumption	30 mW @ 2.5 GHz	0.1 mW @ 2 GHz
Active area	0.03 mm^2^	0.001 mm^2^
